# Inter-laboratory agreement on embryo classification and clinical decision: Conventional morphological assessment vs. time lapse

**DOI:** 10.1371/journal.pone.0183328

**Published:** 2017-08-25

**Authors:** Luis Martínez-Granados, María Serrano, Antonio González-Utor, Nereyda Ortíz, Vicente Badajoz, Enrique Olaya, Nicolás Prados, Montse Boada, Jose A. Castilla, on behalf of Special Interest Group in Quality of ASEBIR (Spanish Society for the Study of Reproductive Biology)

**Affiliations:** 1 U. Reproducción, UGC de Laboratorio Clínico y UGC Obstetricia y Ginecología, Hospital Universitario Virgen de las Nieves, Instituto de Investigación Biosanitaria de Granada (IIBG), Granada, Spain; 2 Clínica IFEM, Córdoba, Spain; 3 Centro MasVida Reproducción, Sevilla, Spain; 4 Instituto Europeo de Fertilidad, Madrid, Spain; 5 Ginefiv, Madrid, Spain; 6 Clínica Tambre, Madrid, Spain; 7 IVI, Sevilla, Spain; 8 Women’s Health Dexeus, Barcelona, Spain; 9 CEIFER Biobanco, Granada, Spain; 10 Departamento de Anatomía y Embriología Humana, Facultad de Medicina, Universidad de Granada, Granada, Spain; Utah State University, UNITED STATES

## Abstract

The aim of this study is to determine inter-laboratory variability on embryo assessment using time-lapse platform and conventional morphological assessment. This study compares the data obtained from a pilot study of external quality control (EQC) of time lapse, performed in 2014, with the classical EQC of the Spanish Society for the Study of Reproductive Biology (ASEBIR) performed in 2013 and 2014. In total, 24 laboratories (8 using EmbryoScope™, 15 using Primo Vision™ and one with both platforms) took part in the pilot study. The clinics that used EmbryoScope™ analysed 31 embryos and those using Primo Vision™ analysed 35. The classical EQC was implemented by 39 clinics, based on an analysis of 25 embryos per year. Both groups were required to evaluate various qualitative morphological variables (cell fragmentation, the presence of vacuoles, blastomere asymmetry and multinucleation), to classify the embryos in accordance with ASEBIR criteria and to stipulate the clinical decision taken. In the EQC time-lapse pilot study, the groups were asked to determine, as well as the above characteristics, the embryo development times, the number, opposition and size of pronuclei, the direct division of 1 into 3 cells and/or of 3 into 5 cells and false divisions. The degree of agreement was determined by calculating the intra-class correlation coefficients and the coefficient of variation for the quantitative variables and the Gwet index for the qualitative variables. For both EmbryoScope™ and Primo Vision™, two periods of greater inter-laboratory variability were observed in the times of embryo development events. One peak of variability was recorded among the laboratories addressing the first embryo events (extrusion of the second polar body and the appearance of pronuclei); the second peak took place between the times corresponding to the 8-cell and morula stages. In most of the qualitative variables analysed regarding embryo development, there was almost-perfect inter-laboratory agreement among conventional morphological assessment (CMA), EmbryoScope™ and Primo Vision™, except for false divisions, vacuoles and asymmetry (users of all methods) and multinucleation (users of Primo Vision™), where the degree of agreement was lower. The inter-laboratory agreement on embryo classification according to the ASEBIR criteria was moderate-substantial (Gwet 0.41–0.80) for the laboratories using CMA and EmbryoScope™, and fair-moderate (Gwet 0.21–0.60) for those using Primo Vision™. The inter-laboratory agreement for clinical decision was moderate (Gwet 0.41–0.60) on day 5 for CMA users and almost perfect (Gwet 0.81–1) for time-lapse users. In conclusion, time-lapse technology does not improve inter-laboratory agreement on embryo classification or the analysis of each morphological variable. Moreover, depending on the time-lapse platform used, inter-laboratory agreement may be lower than that obtained by CMA. However, inter-laboratory agreement on clinical decisions is improved with the use of time lapse, regardless of the platform used.

## Introduction

Precise embryo selection is crucially important to obtaining good results in assisted reproduction techniques. Currently, the two most widespread methods of embryo selection are conventional morphological assessment (CMA) and embryonic morphokinetics using time-lapse (TL) platforms. Inter-laboratory variability in the application of conventional morphology has been demonstrated in embryo classification and in the clinical decision taken [1,2]. In consequence, various scientific societies recommend participation in external quality control (EQC) programmes of embryo evaluation [3,4].

Although TL methods provide a great deal of information on embryo development, their analysis continues to be performed manually [5]. As regards the measurement of times on TL platforms, only one study has evaluated intra- and inter-observer accuracy (among three observers), reporting little variability in this respect [6]. To our knowledge, no study has yet been conducted, or EQC programme implemented, to evaluate inter-laboratory variability in the determination of these times.

Most studies seeking to develop classification algorithms using TL platforms only take into account the timing of embryo development [7–12]. However, these platforms also allow embryos to be selected by evaluating classical morphological characteristics, such as pronuclei features, blastomere size, symmetry, fragmentation and multinucleation [13]. Ahlstrom *et al*. [14] showed that this strategy is clinically more useful than an embryo selection method in which only the timing is analysed. Other authors, using various strategies, have incorporated these characteristics into algorithms based on morphokinetic events [8,15,16]. At present, little is known about the performance of TL in the assessment of morphological characteristics such as fragmentation or multinucleation. Sundvall *et al*. [6], in a study of three observers, obtained a ‘fair’ intraclass correlation coefficient for evenness and multinucleation.

Taking into account the above considerations, the aims of this study are to determine inter-laboratory variability in the assessment of morphokinetic events and morphological characteristics using TL and to compare the inter-laboratory variability in embryo classification and clinical decision after using TL with that observed using CMA. To achieve these study goals, the results of a pilot EQC morphokinetic trial were compared with those obtained by a CMA EQC programme.

## Materials and methods

### External quality control of embryo morphology using time-lapse technology

ASEBIR (Spanish Society for the Study of Reproductive Biology) e-mailed all its members inviting them to participate in a pilot EQC test of embryo morphology using TL technology, informing them that participation would be voluntary, by each laboratory, and that to participate the laboratory must possess and use one of the two platforms evaluated (EmbryoScope™ [Unisense FertiliTech, Denmark] or Primo Vision™ Version 4.3.1.5 [Vitrolife, Sweden]).

In June 2014, each participating laboratory was sent three batches (on a USB device), with videos of the development up to the blastocyst stage of 9–14 embryos from oocytes inseminated by ICSI. Each batch consisted of images of embryos obtained from a different couple. These videos were made from images taken every 5–20 minutes, at different focal planes, of 31 embryos for the users of EmbryoScope™ and of 35 embryos for those of Primo Vision™.

The videos of the embryos used for the trial were obtained from the ICSI programmes at the following ART centres, each of which had the morphokinetic platforms stipulated to be analysed in this study: GineFIV, Madrid; Clínica Tambre, Madrid; Unidad de Reproducción CHU, Granada. The embryos were cultured in vitro, according to the protocol established at each laboratory. All patients signed informed consent authorising the use of anonymised data and images of their embryos for the dissemination of scientific knowledge. This is a retrospective study of anonymised data and images; it therefore does not require the approval of an ethical committee for clinical investigation, according to Spanish law (OrdenSAS/3470/2009).

For the EmbryoScope™ platform, the videos were obtained using the software provided with the system, which generates a TL video from high-definition static images captured every 10 minutes, on seven focal planes. The embryos were cultured at an oxygen concentration of 5%. The TL videos for the Primo Vision™ platform were generated from images consisting of seven 200 μm focal planes, captured every 5 minutes for the first 24 hours and then every 20 minutes until the day of embryo transfer. These embryos were cultured at an oxygen concentration of 21%.

Together with the images, instructions were sent to each participating laboratory with explanations on the operation of the pilot quality control test and a database for recording the times and other morphological characteristics with which the embryos would be classified, in accordance with the ASEBIR criteria [3].

The following morphokinetic events (quantitative variables) were recorded: time elapsed until the extrusion of the second polar body (tPB2), time to the appearance (tPNa) and fading (tPNf) of the pronuclei, time to the first signs of first division (tSt2), time to cell division (t2, t3, t4, t5, t6, t7, t8, t9), time to end of compaction process (tM), time to the first signs of blastulation (tSB), time to full blastocyst stage (tB), time to start of expansion (tE) and time to start of hatching (tHN).

In addition, the following morphological characteristics (qualitative variables) were recorded: number of pronuclei (PN), opposition of PN (yes or no), size of PN (symmetrical or asymmetrical), whether there was a direct division of 1 into 3 cells and/or of 3 into 5 cells, the highest rate of cell fragmentation reached during development (≤10%, >10–25%, >25–36% or >36%), the absence or presence of vacuoles, the asymmetry of blastomeres (symmetry or asymmetry at the 2, 4 or 8 cell stages), multinucleation from the 2-cell stage to the 4-cell stage (absence or presence) and false cleavage (yes or no).

From the morphological characteristics evaluated (cell fragmentation, presence of vacuoles, blastomere asymmetry and multinucleation), together with the number of cells at the time points 16–18 hours (day 1), 43–45 hours (day 2), 67–69 hours (day 3) and 114–118 hours (day 5) after sperm microinjection, the embryos were classified according to the ASEBIR system, using a flow chart to indicate embryo quality and implantation potential, as follows: A—very high; B—high; C—low; D—very low [3].

The laboratories, after classifying the embryos on days 2, 3 and 5 according to the ASEBIR system, had to decide which embryo was optimal on day 5, per batch, for transfer. For the embryos that were not transferred, they also had to decide whether they should be cryopreserved or discarded. The TL users were not asked for a clinical decision on day 2 or 3.

### External quality control programme using conventional morphological assessment

The laboratories that participate in this external quality control program have done so on a voluntary basis, in response to the annual call promoted by ASEBIR since 2003 and which it has organised since 2012 [2,17]. The data analysed in this paper correspond to the 2013–14 editions, in which 39 laboratories took part.

In this programme, the laboratories had online access to a platform, with three batches with videos of zygotes, day 2 and day 3 embryos and two batches with videos of day 4 and day 5 embryos. The optic and magnification used for the recording were Hoffman and 400x, respectively. The recordings were carried out as a daily routine assessment of embryo quality. Each batch consisted of embryo images obtained from different couples. The first three batches were divided into three groups, each one with five videos, of zygotes, day 2 embryos and day 3 embryos, respectively. The last two batches were divided into two groups, one with five videos of day 4 embryos and the second with five videos of day 5 embryos. In the annual ASEBIR external quality control programme, the following morphological characteristics are analysed in day 2 and day 3 embryos: percentage of cell fragmentation, presence or absence of vacuoles, blastomere asymmetry and multinucleation (presence or absence).

The laboratories were asked to classify each day 2, 3 and 5 embryo in accordance with the ASEBIR criteria and to decide, for the last two batches (day 5), which embryo should be transferred. For the last two batches, the laboratories were asked to assume that all embryos were classified as type A at day 3. For the embryos that were not transferred, the laboratories were asked to decide which should be cryopreserved and which should be discarded.

### Statistics methods

For both quality control programmes, a high degree of agreement was considered to have been achieved when over 75% of the laboratories reported the same classification or took the same clinical decision for the embryo.

The data obtained in this trial were analysed with the statistical package SPSS 19.0. For each time analysed, the coefficient of variation (CV) and the intraclass correlation coefficient (ICC), together with the corresponding 95% confidence interval, were calculated. To determine the degree of inter-observer agreement in evaluating the qualitative variables, the AgreeStat2015 [Advanced Analytics, LLC (USA)] program was used to calculate the Gwet AC2 (weighted) concordance index for several observers and several categories, together with the respective 95% confidence intervals. The strength of agreement for the Gwet AC2 statistic was expressed using the following intervals: 0 = no agreement; 0.01–0.20 = slight agreement; 0.21–0.40 = fair agreement; 0.41–0.60 = moderate agreement; 0.61–0.80 = substantial agreement; 0.81–1.00 = almost perfect agreement. The mean times of each event for the embryos analysed with each platform were compared by the Student test. Yates’ chi-square tests were used to compare the categoric data. All tests were conducted at the 5% significance level.

## Results

**Fig 1:**
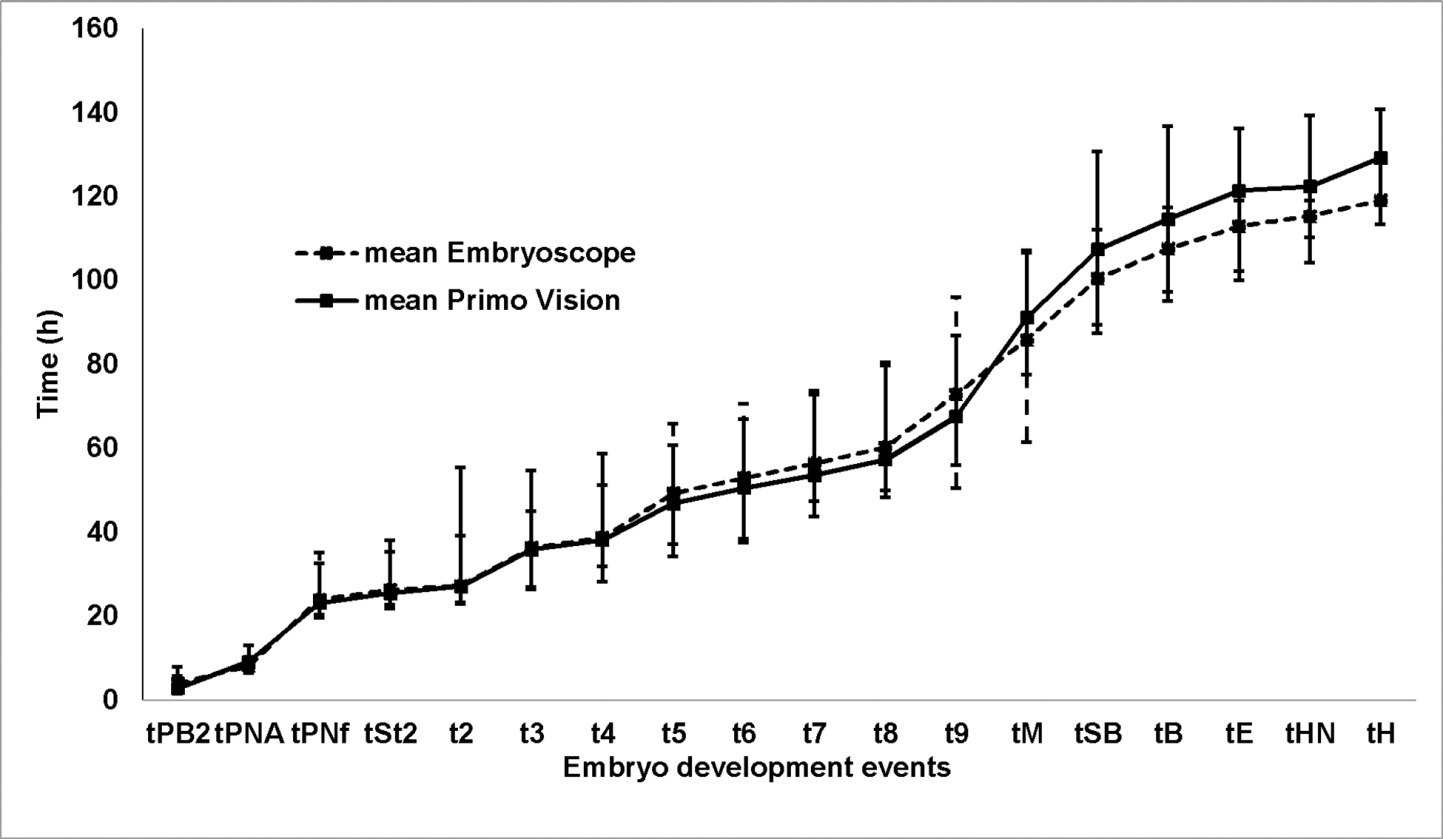
Mean times of the quantitative variables for both morphokinetic platforms (maximum and minimum).

Each of the embryo events analysed with the EmbryoScope™ and Primo Vision™ platforms (Fig 1) presented similar mean times, although from the onset of blastulation these times were rather shorter in the embryos cultured in EmbryoScope™. Raw data are shown in S1–S6 Tables.

**Fig 2:**
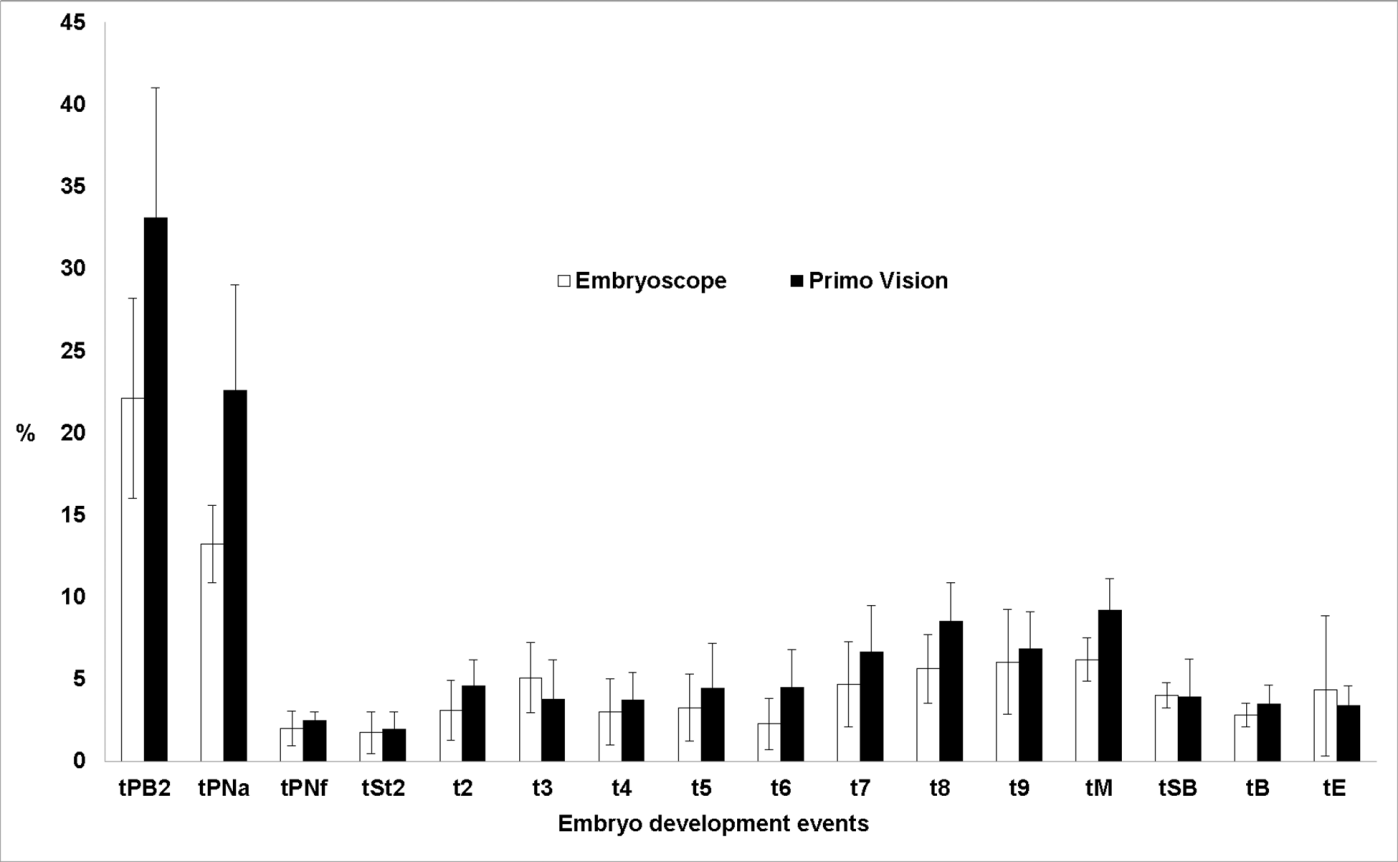
Mean CV and 95% CIs (error bars) obtained at different times of embryo development for both morphokinetic platforms.

**Fig 3:**
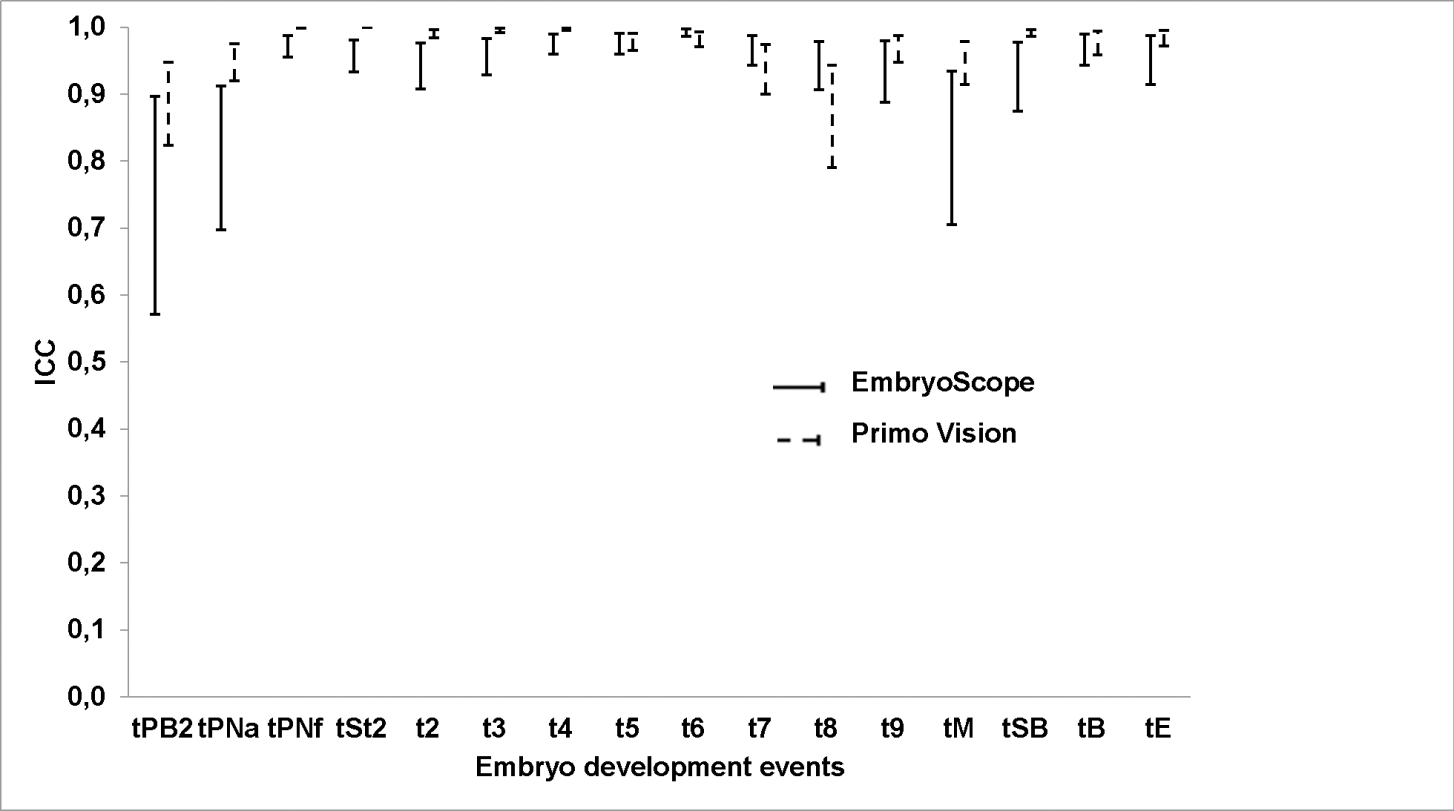
ICC and 95% CIs (error bars) obtained at different times of embryo development, for both morphokinetic platforms.

Both platforms revealed two periods of greater inter-laboratory variability in the timing of embryo development events: first, regarding the initial embryo events (extrusion of the second polar body and the appearance of pronuclei) and then between the 8-cell and morula stages (Figs 2 and 3).

In most of the qualitative variables of embryo development, almost perfect inter-laboratory agreement was observed, both among those using CMA and among those using EmbryoScope™ or Primo Vision™, except for false cleavages, vacuoles and asymmetry, for all the methods analysed, and for multinucleation among the users of Primo Vision™, where the degree of agreement was lower (Table 1).

**Table 1:** Degree of inter-laboratory agreement (Gwet AC2 index) on the evaluation of embryo variables, by TL and CMA.

	Primo Vision^TM^		EmbryoScope^TM^		CMA	
	Gwet	95%CI	Gwet	95%CI	Gwet	95%CI
NumberPN	0.99	0.99 to 1	0.99	0.98 to 1		
SizePN	0.92	0.86 to 0.97	0.97	0.90 to 1		
Opposition2PN	0.89	0.83 to 0.94	0.92	0.86 to 0.97		
Direct division 1 to 3 cells	0.87	0.78 to 0.97	0.88	0.77 to 0.99		
Direct division 3 to 5 cells	0.83	0.74 to 0.93	0.90	0.81 to 1		
Fragmentation	0.83	0.78 to 0.88	0.91	0.87 to 0.95	0.95	0.92 to 0.98
Multinucleation 2–4 cell stage	0.70	0.61 to 0.79	0.94	0.90 to 0.97	0.92	0.86 to 0.98
False cleavage	0.69	0.53 to 0.84	0.77	0.62 to 0.93		
Vacuoles	0.58	0.41 to 0.76	0.57	0.36 to 0.79	0.99	0.99 to 1
Asymmetry	0.39	0.22 to 0.55	0.40	0.25 to 0.54	0.54	0.44 to 0.64

PN: pronuclei

The level of inter-laboratory agreement on embryo classification according to the ASEBIR criteria was similar (moderate-substantial) among users of CMA and of EmbryoScope™, for days 2, 3 and 5. However, there was less agreement (fair-moderate) among the users of Primo Vision™ (Table 2). Similar conclusions were obtained concerning the percentage of embryos presenting a high degree of agreement: the laboratories that used CMA (30.4%, 24/79) and EmbryoScope™ (35.2%, 25/71) obtained similar results, which in both cases were higher than those obtained by the users of Primo Vision™ (20%, 17/85) (Table 3).

**Table 2:** Degree of inter-laboratory agreement (Gwet AC2 index) on embryo classification by ASEBIR criteria and clinical decision.

	Primo Vision^TM^			EmbryoScope^TM^		CMA	
	Gwet	95%CI		Gwet	95%CI	Gwet	95%CI
Classification							
Day 2	0.38	0.24 to 0.52		0.60	0.47 to 0.72	0.64	0.52 to 0.75
Day 3	0.39	0.24 to 0.54		0.56	0.43 to 0.69	0.63	0.49 to 0.78
Day 5	0.59	0.39 to 0.79		0.73	0.60 to 0.86	0.70	0.62 to 0.78
Decision							
Day 5	0.81	0.71 to 0.9		0.87	0.80 to 0.94	0.63	0.54 to 0.72

**Table 3:** Percentage of embryos with a high degree of agreement on embryo classification according to ASEBIR criteria.

	Primo Vision^TM^	EmbryoScope^TM^	CMA
A	18% (5/28)	33% (7/21)	37% (7/19)
B	0% (0/14)	8% (1/13)	9% (2/23)^d^
C	0% (0/18)	19% (4/21)	24% (6/25)^f^
D	48% (12/25)^a^^,^^b^^,^^c^	81% (13/16)^a^^,^^b^^,^^c^^,^^e^	75% (9/12)^a^^,^^b^^,^^c^

^a^ p ≤ 0.05 A vs D

^b^ p ≤ 0.05 B vs D

^c^ p ≤ 0.05 C vs D

^d^ p ≤ 0.05 A vs B

^e^ p ≤ 0.05 Primo Vision vs EmbryoScope

^f^ p ≤ 0.05 Primo Vision vs CMA

The percentage of embryos presenting a high degree of inter-laboratory agreement when classified according to ASEBIR criteria, both with CMA and with the TL platforms, was higher when the embryo was classified as very low quality (type D) by the majority of the participating centres, than for categories A, B and C (Table 3).

With respect to the clinical decision, at day 5, the level of agreement on the clinical decision was higher among the users of TL (almost perfect) than among the laboratories using CMA (moderate) (Table 2). In addition, the percentage of embryos for which there was a high level of agreement regarding the clinical decision was higher among users of the TL platforms than among CMA users (Table 4).

**Table 4:** Percentage of embryos with a high degree of agreement on clinical decision.

	Primo Vision^TM^	EmbryoScope^TM^	CMA
Transfer	0% (0/3)	67% (2/3)	33% (1/3)
Cryopreserve	42% (5/12)	60% (9/15)	20% (3/15)
Discard	85% (17/20)	100% (13/13)	50% (1/2)
Overall	62% (22/35)^a^	77% (24/31)^a^	25% (5/20)

^a^ p ≤ 0.05 Time Lapse vs CMA

The percentage of embryos presenting a high level of inter-laboratory agreement regarding the clinical decision, both with CMA and with the TL platforms, was higher for the embryos that were to be discarded than for those to be transferred (Table 4).

## Discussion

In evaluating an embryo, the observer factor is one of the most important points, since the subjective component–observation of images of the embryo–is inherent to the assessment. In theory, with platforms based on morphokinetics, this factor should be attenuated, due to the greater information thus obtained and the possibility of viewing the images as many times as necessary without prejudice to the status of the embryo. However, according to Lundin *et al*. [18], the fact that cell development events can be defined by the starting point, by the mid-event status or by the last photogram observed could hamper the comparison of results obtained by different laboratories. Our results seem to confirm this hypothesis, as events that take longer to occur (30–45 min.) such as the extrusion of the second polar body or the appearance of pronuclei, coincide with greatest inter-laboratory variability. By contrast, in events that occur almost instantaneously, such as cell division or the disappearance of pronuclei, the inter-laboratory variability observed was significantly lower.

The other peak of inter-laboratory variability observed in our study took place following the 8-cell stage, possibly because the cells are then in greater proximity, making it difficult to count the blastomeres as the compaction process begins. Furthermore, this compaction may be only partial, such that some cells are excluded, making it impossible to distinguish clearly whether the cells can still be counted or whether the morula stage has begun.

The systematic review by Kirkegaard *et al*. [19], on the morphokinetic parameters related to achieving pregnancy or embryo euploidy, concluded that the only parameters with predictive capacity, presented by more than one laboratory, were outside the periods of high inter-laboratory variability discussed above. This suggests that the parameters for these periods of high inter-laboratory variability have little external validity and should not be used in embryo classification algorithms. In fact, one of the most commonly used algorithms, that of Meseguer *et al*. [7], updated by Basile *et al*. [10], does not include any of the parameters of high inter-observer variability described in the present study.

The high degree of agreement observed with respect to morphokinetic events coincides with the findings of Sundvall *et al*. [6]. In our study, the validity of this conclusion was strengthened by the participation of 24 laboratories, compared to the three observers at the single laboratory studied by Sundvall *et al*. [6].

There was no improvement in the level of inter-laboratory agreement between users of TL technology and of CMA, for any independent morphological characteristic or in embryo classification according to ASEBIR criteria. This may be because the criteria for assessing morphological characteristics, which are often transient, are not well defined [20,21]. This means that although there is a lot of information, there are no clear criteria for its assessment. In addition, it is noteworthy that among the users of Primo Vision™ the level of agreement was significantly lower concerning fragmentation and multinucleation than among those of EmbryoScope™ and CMA. This may be due to technical (optical) limitations of this platform, hampering the evaluation of these characteristics. This finding corroborates previous recommendations [18,22] that any new embryo selection technology should be assessed prior to its implementation in assisted reproduction laboratories. In this respect, our results show that the key performance index depends on the technology used and on the laboratory that uses it.

It is noteworthy that the absence of any improvement in the level of inter-observer agreement among users of TL extends to the evaluation of asymmetry, an area in which a lower level of agreement among CMA users is to be expected, since the users of CMA are not equipped with the accurate instruments needed to evaluate cell asymmetry. In contrast, many morphokinetic platforms do have these instruments. Our results could be explained by reference to three factors. First, not all laboratories use these instruments. Second, not all of them employ a clear-cut definition of embryonic asymmetry. Finally, the above-mentioned transient nature of the morphological characteristics, including asymmetry, would hinder their analysis [23].

Kirkegaard *et al*. [19] suggested that embryo classification should be used for ranking rather than selection, to avoid the risk of discarding usable embryos, taking into account that no model has yet demonstrated 100% sensitivity and specificity in the prediction of implantation potential. Indeed, Mastenbroek *et al*. [24], in view of this risk, questioned the need for any embryo selection at all and recommended, at least, the redefinition of embryo selection. To minimise this negative effect of embryo selection, it is crucial to control the accuracy of the embryo selection criteria, which, as our results show, vary according to the technology used.

Unlike in embryo classification, the level of agreement on the clinical decision is higher with TL platforms than with CMA. This result, may be due to the high degree of agreement observed in qualitative variables, which can only be observed by TL [25,26], and that have been related to poor embryo quality [27–29]. However, the level of agreement on the variables that can be observed with both methods is lower than that found in the above-described case. This would appear to be a logical outcome, since in their decision-making CMA users only use variables for which there is a lower level of agreement; hence, the clinical decision based on these variables presents a lower level of agreement than when other variables are also taken into consideration (as is the case at laboratories that use TL).

Some authors have suggested that the use of algorithms based on morphokinetic events should be complemented by the observation of classical morphological characteristics by TL [23] or by CMA [30]. However, our results show that this practice remains subject to inter-laboratory variability. The use of TL technology to evaluate classical morphological variables requires these variables to be clearly defined beforehand. For example, the degree of fragmentation to be taken into account can be assessed in various ways: as the degree of maximum fragmentation reached during the entire process of embryo development; as the degree of fragmentation in the last photogram obtained before each cell cycle; or as the degree of fragmentation on the day of the transfer [14]. According to our data, the use of morphokinetic platforms does not guarantee a higher level of inter-laboratory agreement in the assessment of morphological variables than when these same variables are evaluated by CMA. Therefore, we believe that before recommending the incorporation of these variables into the corresponding algorithms, the definition of parameters such as asymmetry, multinucleation and vacuoles, under TL technology, must be standardised.

The results of this study show that assisted reproduction laboratories tend to be in closer agreement on the evaluation of low-quality embryos than of high-quality ones, and of embryos that are to be discarded than on those to be transferred, independently of the embryo evaluation technique used, as reported previously by Arce *et al*. [1] and Castilla *et al*. [2] for the CMA method. The high degree of accuracy observed in the classification of low-quality embryos would produce a low rejection rate of viable embryos. On the other hand, the heterogeneity of results observed in the classification of high-quality embryos makes it difficult to obtain reproducible models that would enable a substantial increase in implantation rates [31]. We believe that these factors could be behind the slow incorporation of time-lapse platforms into clinical practice.Our study has several limitations. The embryos evaluated were different in each method and subjected to different culture conditions. This could lead to differences both in embryo development and in embryo morphology, which could make the laboratories more likely to coincide in their assessments with one method than the other. However, the development times of the embryos analysed were similar for each of the platforms and although no significant differences were obtained, from the morula stage onwards there was a decrease in the rate of development for the embryos examined using the Primo Vision™ system. This may be due to the fact that the embryos in the EmbryoScope™ system were cultured in an atmosphere with 5% oxygen, while those cultured in the Primo Vision™ system had an atmosphere with 21% oxygen. This circumstance would be related to previous reports [32,33] that embryo development is faster when it takes place at low oxygen concentrations.

Another limitation is that it would have been preferable to know the implantation rates of the embryos evaluated and to determine whether the inter-laboratory variability on the clinical decision and on embryo quality influenced the effectiveness of assisted reproduction cycles. Furthermore, in this study, no analysis was made of intra-observer agreement, although Sundvall *et al*. [6] previously reported that this parameter is low, in a similar situation. Finally, the possibility of participation bias cannot be ruled out. As participation in the trial was voluntary, the participating laboratories may have been those which were most motivated and aware of the importance of quality management. Therefore, the inter-laboratory variability results obtained may not represent those actually present in all the centres that were invited to participate, i.e. our results could be under-estimates.

In view of the findings of this study, we conclude that it is necessary to develop guidelines to define and interpret the classical morphological characteristics observed by TL before incorporating these characteristics into diagnostic algorithms based on TL.

## Supporting information

S1 Table Mean times and standard deviations of the morphokinetic events of the embryos analysed with EmbryoScope. B: Batch, O: Oocyte.
(PDF)

S2 Table Mean times and standard deviations of the morphokinetic events of the embryos analysed with Primo Vision. B: Batch, O: Oocyte.
(PDF)

S3 Table Morphological characteristics and clinical decision of the embryos analysed with EmbryoScope. Majority response (percentage of centres giving the majority response). PN:Pronuclei, NC:No consensus.
(PDF)

S4 Table Morphological characteristics and clinical decision of the embryos analysed with Primo Vision. Majority response (percentage of centres giving the majority response). PN:Pronuclei, NC:No consensus.
(PDF)

S5 Table Morphological characteristics of the Day 2 and Day 3 embryos analysed with CMA. Majority response (percentage of centres giving the majority response).NC: No consensus.
(PDF)

S6 Table Morphological classification and clinical decision of the Day 5 embryos analysed with CMA. Majority response (percentage of centres giving the majority response).
(PDF)
