# Supplementary material for: Inter-laboratory agreement on embryo classification and clinical decision: Conventional morphological assessment vs. time lapse
Source: PLoS One. 2017 Aug 25;12(8):e0183328. doi: 10.1371/journal.pone.0183328 (PMC5571938; doi:10.1371/journal.pone.0183328)
Supplement: S1 Table — B: Batch, O: Oocyte. (PDF) [file pone.0183328.s001.pdf]

S1 Table. Mean times and standard deviations of the morphokinetic events of the embryos analysed with EmbryoScope.

EmbryoScope	tPB2	tPNa	tPNf	tSt2	t2	t3	t4	t5	t6	t7
B1O1	5,7 ±1,9	9,7 ±0,6	29,1 ±0,2	32,0 ±0,6	34,3 ±2,6	44,9 ±4,5	45,0 ±4,6	51,2 ±6,1	53,9 ±7,0	61,0 ±15,1
B1O2	5,0 ±2,3	6,5 ±0,7	20,4 ±0,3	22,7 ±0,3	23,6 ±0,6	36,0 ±2,7	36,0 ±0,6	47,5 ±0,2	47,7 ±0,3	48,3 ±0,8
B1O3	5,4 ±1,4	8,4 ±1,6	26,6 ±0,3	29,0 ±0,1	30,1 ±0,9	44,9 ±0,4	46,0 ±0,8	63,0 ±0,0	63,5 ±0,7	65,9 ±1,4
B1O4	4,8 ±2,2	7,3 ±1,2	23,4 ±0,2	25,3 ±0,2	25,7 ±0,3	36,8 ±0,3	37,4 ±0,2	52,8 ±0,2	55,6 ±0,3	63,8 ±4,3
B1O5										
B1O6	3,7 ±0,3	8,1 ±1,1	23,8 ±0,4	25,9 ±0,3	26,6 ±0,4	40,2 ±1,7	41,4 ±0,2	65,8 ±0,3	68,6 ±0,3	68,8 ±0,4
B1O7	3,7 ±0,3	7,4 ±0,8	22,8 ±0,4	24,8 ±0,7	26,0 ±1,2	34,7 ±3,1	36,6 ±0,8	49,9 ±0,3	50,5 ±0,0	50,5 ±0,2
B1O8	4,1 ±1,2	7,8 ±1,9	24,1 ±0,4	26,1 ±0,4	29,5 ±3,8	34,9 ±4,7	37,7 ±0,3	52,4 ±0,2	52,8 ±0,2	54,6 ±0,2
B1O9	4,0 ±0,4	8,0 ±1,3	24,6 ±0,3	26,7 ±0,6	29,1 ±3,0	37,5 ±0,3	38,1 ±1,1	52,0 ±0,0	52,0 ±0,1	53,3 ±1,2
B1O10	3,8 ±0,0	8,3 ±1,0	22,6 ±0,3	24,8 ±0,2	25,2 ±0,3	36,6 ±0,6	37,3 ±0,0	38,9 ±4,4	50,8 ±0,5	52,4 ±0,8
B1O11	3,8 ±0,0	7,3 ±0,5	23,1 ±3,6	26,6 ±4,6	31,6 ±6,3	42,6 ±5,3	47,5 ±2,3	50,8 ±7,3	70,6 ±0,6	73,7 ±3,8
B1O12	5,4 ±3,2	8,0 ±0,9	35,1 ±0,2	38,0 ±1,0	39,1 ±0,3	39,7 ±0,6	51,2 ±4,8	53,4 ±0,3	65,3 ±10,0	70,8 ±7,5
B2O1										
B2O2	4,7 ±0,8	7,9 ±1,1	22,9 ±0,3	25,1 ±0,1	25,5 ±0,4	34,7 ±3,2	37,6 ±1,0	49,6 ±0,2	50,4 ±0,4	50,0 ±3,6
B2O3	2,7 ±0,4	7,9 ±1,0	21,0 ±0,5	22,8 ±0,1	23,2 ±0,2	34,3 ±0,1	34,7 ±0,3	46,4 ±0,2	48,8 ±0,2	49,2 ±0,3
B2O4	2,9 ±0,5	8,0 ±1,5	23,6 ±0,3	25,7 ±0,2	26,1 ±0,1	38,2 ±0,2	38,3 ±0,2	53,7 ±0,4	54,5 ±0,3	55,2 ±0,2
B2O5	3,9 ±1,1	9,4 ±2,0	21,4 ±0,4	23,7 ±0,2	24,8 ±1,3	30,7 ±5,5	34,8 ±4,3	46,6 ±4,4	50,6 ±0,4	58,0 ±9,3
B2O6										
B2O7	4,0 ±0,6	9,8 ±2,2	24,1 ±0,2	26,3 ±0,1	26,7 ±0,4	26,9 ±0,3	31,9 ±7,3	37,1 ±0,2	38,4 ±0,4	51,6 ±5,4
B2O8	3,8 ±1,1	7,9 ±1,4	23,3 ±0,4	24,6 ±0,5	25,8 ±0,3	28,9 ±3,4	37,4 ±0,2	39,1 ±3,6	47,9 ±4,3	55,8 ±1,8
B2O9	5,1 ±2,6	8,7 ±1,3	22,7 ±0,4	24,6 ±0,3	25,0 ±0,5	35,3 ±0,2	36,0 ±0,1	48,4 ±0,2	48,7 ±1,3	52,4 ±1,6
B2O10	5,4 ±3,2	8,0 ±0,9	35,1 ±0,2	38,0 ±1,0	39,1 ±0,3	39,7 ±0,6	51,2 ±4,8	53,4 ±0,3	65,3 ±10,0	70,8 ±7,5
B3O1	3,4 ±0,4	8,7 ±0,7	23,6 ±0,3	25,2 ±0,4	26,0 ±0,1	37,3 ±0,1	37,6 ±0,2	49,4 ±0,2	52,8 ±0,2	53,2 ±0,3
B302	3,0 ±0,5	6,9 ±0,4	23,8 ±0,4	26,2 ±0,2	26,7 ±0,2	30,3 ±2,8	37,0 ±0,5	38,7 ±4,2	48,9 ±0,5	49,7 ±0,8
B3O3	5,0 ±1,8	7,9 ±1,0	24,0 ±0,2	26,5 ±0,2	27,1 ±0,2	39,2 ±0,2	42,1 ±0,1	51,0 ±0,0	55,0 ±3,6	65,5 ±0,3
B3O4										
B3O5	3,9 ±0,5	9,1 ±0,7	23,6 ±0,7	26,0 ±0,2	26,3 ±0,2	37,5 ±0,2	38,1 ±0,2	50,3 ±0,1	50,7 ±0,3	52,0 ±0,0
B306	4,5 ±1,2	9,6 ±1,7	24,6 ±0,6	26,3 ±0,5	27,1 ±0,2	33,2 ±3,7	37,4 ±0,9	44,0 ±6,3	50,5 ±1,2	55,7 ±11,4
B307	3,6 ±1,3	8,0 ±2,0	22,1 ±0,3	24,8 ±0,2	25,3 ±0,1	33,6 ±3,7	35,3 ±0,2	46,7 ±0,0	46,7 ±0,3	47,5 ±0,3
B3O8	3,9 ±0,3	8,2 ±0,5	25,9 ±0,5	28,3 ±0,0	28,5 ±0,2	39,3 ±0,2	39,7 ±0,3	52,5 ±0,3	53,0 ±0,0	53,1 ±0,3
B3O9	3,9 ±0,5	8,2 ±0,6	24,4 ±0,8	27,2 ±0,3	27,6 ±0,2	38,6 ±0,2	39,0 ±0,0	52,1 ±0,2	52,7 ±0,2	53,6 ±0,3

S1 Table. Mean times and standard deviations of the morphokinetic events of the embryos analysed with EmbryoScope (continuation).

EmbryoScope	t8	t9	tM	tSB	tB	tE	tHN	tH
B1O1	66,1 ±9,6	78,0 ±12,7	107,1	110,2 ±2,7				
B1O2	50,0 ±1,1	63,8 ±10	82,6 ±2,3	95,0 ±5,5	105,0 ±6,0	105,0 ±35,9	118,0	
B1O3	70,1 ±2,0	96,0 ±0,3	96,7 ±5,5	107,5 ±4,6	117,4 ±0,8			
B1O4	72,3 ±8,2	89,4 ±1,0	86,1 ±11,1	106,9 ±3,1				
B1O5								
B1O6	79,6 ±12,7		83,8 ±2,5	97,3 ±5,2	112,1			
B1O7	51,3 ±0,2	73,0 ±0,5	81,2 ±1,8	87,5 ±5,0	97,3 ±4,5	106,6 ±5,2	110,2 ±4,8	
B1O8	60,7 ±0,4		72,5 ±5,4	95,2 ±7,8	107,0 ±4,0	115,1 ±2,0	113,7 ±2,1	
B1O9	56,6 ±1,2	72,3 ±10,4	81,8 ±3,9	91,1 ±3,0	99,0 ±4,7	111,3 ±4,7	113,2 ±4,1	
B1O10	55,6 ±1,7	59,0 ±0,8	86,5 ±11,8	112,0 ±4,2	117,5			
B1O11	80,4 ±1,1	84,2 ±5,0	91,5 ±3,2	105,1 ±0,2				
B1O12	75,7 ±1,2	76,8 ±0,2	101,5 ±5,6	103,9 ±0,5				
B2O1								
B2O2	52,9 ±3,0	71,3 ±0,5	81,6 ±5,6	93,3 ±4,1	98,9 ±4,5	106,4 ±2,8	111,8 ±3,9	119,5 ±0,0
B2O3	55,8 ±7,1	64,2 ±6,1	81,2 ±6,2	97,9 ±3,3	103,8 ±2,3	110,8 ±2,1		
B2O4	57,7 ±6,7	73,4 ±1,7	82,3 ±2,1	98,6 ±5,2	109,6 ±3,5	115,5 ±1,9	117,0	
B2O5	63,7 ±1,8	73,0 ±0,4	89,8 ±4,8	100,9 ±7,3	111,9 ±2,2	119,1 ±1,0		
B2O6								
B2O7	49,9 ±0,5	50,5 ±0,7	61,5 ±3,3					
B2O8	57,3 ±2,3	65,5 ±12,9	86,8 ±3,1					
B2O9	58,7 ±5,9	72,5 ±8,3	83,8 ±4,9	97,2 ±4,3	102,8 ±2,5	110,8 ±3,6	113,6 ±2,8	118,7 ±1,0
B2O10	75,7 ±1,2	76,8 ±0,2	101,5 ±5,6	103,9 ±0,5				
B3O1	54,2 ±0,1	73,6 ±0,8	82,8 ±2,4	101,3 ±3.8	109,7 ±3,6	116,8 ±1,5		
B302	50,8 ±0,8	57,9 ±9,5	84,4 ±8,8	99,1 ±3,5	105,4 ±2,9	114,0 ±4,2	116,0	
B3O3	67,6 ±0,3	71,4 ±0,5	86,6 ±9,3	105,3 ±4,2	114,2 ±3,7			
B3O4								
B3O5	54,5 ±5,8	69,6 ±2,0	81,8 ±4,7	102,0 ±4,5	111,7 ±4,0	118,4 ±0,5		
B306	68,7 ±8,2	79,2 ±24,8	100,8 ±7,7	108,1 ±1,6				
B307	50,1 ±6,7	66,6 ±0,2	80,9 ±8,4	94,7 ±1,9	101,0 ±2,9	113,4 ±3,3	115,1	
B3O8	54,8 ±0,3	75,7 ±0,5	87,1 ±5,2	99,7 ±3,0	106,8 ±2,4	115,1 ±1,9	116,2 ±1,5	
B3O9	54,0 ±0,5	74,5 ±0,5	89,5 ±6,7	101,2 ±5,4	108,5 ±1,5	114,9 ±2,2	115,9 ±1,1	
