# Supplementary material for: Inter-laboratory agreement on embryo classification and clinical decision: Conventional morphological assessment vs. time lapse
Source: PLoS One. 2017 Aug 25;12(8):e0183328. doi: 10.1371/journal.pone.0183328 (PMC5571938; doi:10.1371/journal.pone.0183328)
Supplement: S2 Table — B: Batch, O: Oocyte. (PDF) [file pone.0183328.s002.pdf]

S2 Table. Mean times and standard deviations of the morphokinetic events of the embryos analysed with Primo Vision.

Primo Vision	tPB2	tPNa	tPNf	tSt2	t2	t3	t4	t5	t6	t7
B1O1	2,8 ±0,5	8,6 ±1,4	23,8 ±1,7	26,0 ±1,9	27,0 ±1,4	38,3 ±1,1	39,7 ±1,4	50,5 ±1,0	50,9 ±1,0	52,0 ±1,4
B1O2										
B1O3	1,9 ±0,6	10,7 ±1,6	21,0 ±0,2	23,3 ±0,2	23,9 ±0,4	34,7 ±0,1	35,2 ±0,6	48,0 ±0,1	48,6 ±0,4	49,3 ±0,9
B1O4	2,6 ±0,4	6,9 ±1,0	25,2 ±0,3	27,4 ±0,2	27,8 ±0,2	31,4 ±2,0	39,4 ±0,4	41,4 ±2,0	50,9 ±1,4	53,8 ±1,7
B1O5										
B1O6	2,4 ±0,4	12,4 ±3,0	22,1 ±0,4	25,0 ±0,2	25,9 ±0,7	38,1 ±0,1	38,8 ±0,7	51,5 ±0,1	52,0 ±0,3	52,8 ±1,0
B1O7	2,2 ±0,4	6,9 ±1,7	20,9 ±0,4	23,6 ±0,2	24,3 ±0,2	36,1 ±0,5	37,5 ±0,8	39,4 ±2,9	49,9 ±0,3	52,8 ±7,6
B1O8	2,5 ±0,5	9,8 ±1,6	20,9 ±1,2	23,7 ±0,2	24,2 ±0,3	26,4 ±0,2	28,2 ±1,5	40,5 ±0,7	43,1 ±0,9	54,9 ±8,7
B1O9	2,2 ±1,0	6,7 ±2,3	22,2 ±0,3	24,5 ±0,2	25,4 ±0,7	28,6 ±0,6	38,2 ±0,3	39,1 ±0,5	52,1 ±0,7	54,2 ±0,2
B1O10	2,9 ±0,5	8,5 ±1,5	22,2 ±0,6	24,3 ±0,2	24,8 ±0,2	27,3 ±0,7	36,4 ±1,0	37,5 ±1,7	41,1 ±2,7	47,0 ±3,3
B1O11	3,2 ±3,6	11,7 ±3,3	32,2 ±0,6	35,0 ±0,2	36,0 ±1,0	54,7 ±0,6	58,8 ±o,4	60,7 ±1,9	63,6 ±9,5	64,2 ±11,1
B1O12	2,8 ±1,5	10,3 ±3,4	23,1 ±0,5	25,3 ±0,1	27,4 ±3,4	37,7 ±0,1	38,3 ±0,6	51,7 ±0,1	54,5 ±6,6	54,7 ±6,4
B1O13	3,3 ±1,2	8,1 ±1,3	23,6 ±0,6	26,2 ±0,1	26,9 ±0,8	40,3 ±0,1	41,3 ±0,7	46,1 ±5,9	54,1 ±4,6	56,7 ±0,8
B1O14	3,2 ±0,6	9,1 ±2,5	24,0 ±0,5	26,0 ±0,3	27,0 ±0,9	39,1 ±1,4	40,8 ±4,1	52,3 ±0,5	54,2 ±0,7	56,5 ±3,6
B2O1	3,2 ±0,6	9,1 ±2,5	24,0 ±0,5	26,0 ±0,3	27,0 ±0,9	39,1 ±1,4	40,8 ±4,1	52,3 ±0,5	54,2 ±0,7	56,5 ±3,6
B2O2	2,2 ±0,7	7,7 ±1,7	22,8 ±0,4	26,5 ±3,6	27,9 ±3,4	36,9 ±4,0	40,5 ±3,7	52,4 ±6,1	58,0 ±6,4	61,3 ±6,2
B2O3										
B2O4	3,0 ±2,2	10,1 ±2,4	22,0 ±0,5	24,3 ±0,2	24,7 ±0,3	37,4 ±5,0	43,1 ±7,0	55,4 ±8,2	62,1 ±10,1	72,9 ±12,8
B2O5	3,2 ±1,3	9,1 ±1,9	24,1 ±0,6	26,0 ±0,4	27,1 ±1,6	36,8 ±2,6	38,3 ±0,6	48,0 ±6,4	53,2 ±0,7	62,6 ±10,9
B2O6	3,2 ±0,6	9,1 ±2,5	24,0 ±0,5	26,0 ±0,3	27,0 ±0,9	39,1 ±1,4	40,8 ±4,1	52,3 ±0,5	54,2 ±0,7	56,5 ±3,6
B2O7	3,3 ±0,8	9,1 ±2,1	23,2 ±0,7	25,6 ±0,5	26,4 ±0,8	37,3 ±0,2	38,3 ±1,0	50,5 ±0,1	50,8 ±0,2	51,2 ±0,5
B2O8	2,6 ±1,8	12,5 ±2,9	22,9 ±0,7	27,5 ±0,3	28,2 ±1,1	40,1 ±0,3	41,4 ±0,7	53,1 ±0,6	67,0 ±5,1	71,7 ±4,3
B2O9	3,4 ±1,1	9,5 ±2,2	19,6 ±0,5	21,8 ±0,5	22,8 ±1,5	32,6 ±0,2	33,8 ±0,7	42,3 ±2,2	44,9 ±1,1	47,5 ±3,0
B2O10	2,2 ±0,6	7,9 ±1,3	19,7 ±0,3	21,9 ±0,2	23,1 ±2,3	32,0 ±2,3	32,9 ±0,2	34,3 ±0,6	37,5 ±4,3	43,9 ±3,8
B2O11	3,9 ±1,0	9,0 ±1,5	25,0 ±0,3	27,0 ±0,5	28,1 ±0,3	30,7 ±3,3	35,2 ±5,2	37,6 ±9,7	41,8 ±3,6	43,7 ±11,0
B2O12	3,2 ±2,0	12,4 ±1,8	23,4 ±0,5	25,4 ±0,1	26,4 ±2,0	34,9 ±4,5	37,1 ±2,7	44,0 ±7,1	49,6 ±5,4	52,2 ±3,8
B3O1	2,2 ±0,8	7,8 ±1,5	19,9 ±1,3	22,5 ±1,0	24,1 ±1,8	35,9 ±0,2	36,5 ±0,6	48,2 ±0,9	49,1 ±1,1	50,3 ±1,5
B3O2	2,7 ±0,6	6,9 ±1,3	23,5 ±0,5	25,3 ±0,3	27,0 ±2,0	36,5 ±0,3	38,0 ±1,1	47,6 ±0,4	48,5 ±0,7	50,9 ±4,4
B3O3	2,5 ±0,8	7,6 ±1,4	20,9 ±0,4	22,8 ±0,2	23,7 ±1,1	34,6 ±0,1	35,2 ±0,9	46,1 ±0,2	46,9 ±0,5	47,6 ±0,8
B3O4	3,3 ±0,9	10,6 ±1,9	21,1 ±0,4	23,0 ±0,2	24,1 ±1,3	34,2 ±0,1	34,6 ±0,5	45,4 ±0,2	46,1 ±0,3	46,9 ±0,6
B305	2,3 ±0,6	9,0 ±1,1	20,4 ±0,6	22,3 ±0,2	23,3 ±1,1	33,9 ±0,1	34,6 ±0,7	44,5 ±0,2	44,8 ±0,2	45,6 ±0,8
B3O6	2,3 ±0,8	10,4 ±2,1	23,7 ±0,5	25,3 ±0,4	26,2 ±0,6	37,5 ±0,2	38,5 ±0,9	50,2 ±0,1	51,0 ±1,0	53,4 ±2,0
B3O7										
B3O8	2,4 ±0,9	8,7 ±1,4	25,3 ±0,5	27,2 ±0,1	28,1 ±0,7	38,6 ±0,1	39,1 ±0,7	51,3 ±0,4	51,8 ±0,3	52,4 ±0,7
B3O9	4,6 ±0,3	13,0 ±6,8	32,6 ±0,4	35,4 ±1,0	55,5 ±5,3					

S2 Table. Mean times and standard deviations of the morphokinetic events of the embryos analysed with Primo Vision (continuation).

Primo Vision	t8	t9	tM	tSB	tB	tE	tHN	tH
B1O1	55,3 ±6,0	71,7 ±6,6	89,8 ±8,7	107,8 ±3,9	115,4 ±5,3	120,8 ±8,1	129,0 ±2,9	133,8
B1O2								
B1O3	51,0 ±1,8	62,1 ±3,1	86,9 ±8,6	107,3 ±11,3	130,0 ±5,6	136,1 ±2,4	138,0 ±0,7	140,8
B1O4	55,4 ±2,4	56,6 ±3,0	96,9 ±15,8	117,7 ±11,4				
B1O5								
B1O6	56,4 ±7,6	78,9 ±0,6	105,7 ±14,3	130,7 ±4,7	136,8 ±1,6			
B1O7	53,1 ±1,1	55,9 ±4,1	88,7 ±5,4	107,0 ±2,9	108,8 ±10,9	116,3 ±2,6	116,4 ±1,4	123,4
B1O8	64,2 ±2,1	68,3 ±3,2	95,6 ±13,3					
B1O9	55,1 ±1,2	59,8 ±7,6	96,9 ±7,8	111,9 ±1,1	121,2 ±5,5	130,2 ±7,4		
B1O10	50,8 ±3,6	56,9 ±7,4	97,9 ±9,0	111,0 ±3,0	118,0 ±5,4	126,3 ±12,7		
B1O11	67,0 ±10,3	74,2 ±1,2	104,5 ±2,0	110,1 ±1,5	117,3 ±3,0	120,7 ±0,5	121,6 ±1,0	
B1O12	57,3 ±7,6	70,2 ±6,4	86,0 ±12,9	100,5 ±29,1	116,9 ±4,0	120,7 ±2,6	121,4 ±1,0	
B1O13	59,7 ±5,5	70,6 ±8,7	97,7 ±10,7	114,2 ±2,0	118,8 ±3,3	126,9 ±6,4		
B1O14	61,2 ±6,3	70,5 ±7,9	89,7 ±9,5	106,6 ±2,3	113,4 ±4,6	121,1 ±2,4	125,2 ±1,8	
B2O1	61,2 ±6,3	70,5 ±7,9	89,7 ±9,5	106,6 ±2,3	113,4 ±4,6	121,1 ±2,4	125,2 ±1,8	
B2O2	63,3 ±2,3	73,1 ±5,7	86,4 ±13,5	107,5 ±3,0	118,4 ±8,8	132,5 ±6,9		
B2O3								
B2O4	79,7 ±14,1	81,8 ±16,2	106,6 ±2,5	122,7 ±0,3	125,8			
B2O5	65,5 ±11,6	75,3 ±4,0	93,2 ±7,4	112,6 ±3,3	118,9 ±3,8	125,7 ±3,3	133,4 ±0,4	
B2O6	61,2 ±6,3	70,5 ±7,9	89,7 ±9,5	106,6 ±2,3	113,4 ±4,6	121,1 ±2,4	125,2 ±1,8	
B2O7	56,0 ±7,3	70,0 ±1,5	91,2 ±8,4	111,3 ±1,9	117,6 ±1,9	126,4 ±7,1	118,8	
B2O8	79,4 ±3,9	86,9 ±6,6	100,2 ±3,1					
B2O9	51,6 ±6,0	58,5 ±4,3	84,6 ±6,4	101,3 ±1,0	106,5 ±3,8	115,1 ±2,5		
B2O10	48,3 ±6,9	57,2 ±10,3	84,8 ±10,2	107,0 ±2,9	113,7 ±5,1	128,8 ±8,3	137,8 ±0,8	139,2
B2O11	50,2 ±8,8	57,3 ±10,6	84,8 ±11,1	100,0 ±6,7	111,4 ±8,5	125,7 ±6,3	129,7 ±1,4	139,2
B2O12	54,0 ±1,0	63,4 ±10,5	89,8 ±6,1	105,9 ±2,2	115,2 ±3,4	117,4 ±0,4		
B3O1	53,2 ±2,6	65,3 ±4,0	95,2 ±15,7	117,0 ±8,7	125,7 ±9,8	136,3 ±6,9	139,2 ±1,8	140,8
B3O2	56,0 ±6,4	63,9 ±4,0	88,3 ±13,1	112,2 ±4,7	121,5 ±2,4	131,9 ±4,1		
B3O3	52,8 ±7,2	64,8 ±1,2	77,6 ±4,4	92,3 ±1,0	98,7 ±2,9	105,0 ±4,5	105,9 ±4,3	114,8
B3O4	50,2 ±5,9	65,2 ±0,6	77,9 ±5,2	89,3 ±1,5	95,0 ±3,1	102,3 ±2,7	104,2 ±2,8	113,5
B305	50,5 ±8,7	69,4 ±1,0	86,7 ±6,7	103,1 ±1,1	108,2 ±2,6	116,2 ±4,3	126,8 ±1,3	
B3O6	56,5 ±6,3	71,4 ±2,2	86,6 ±5,2	96,2 ±0,8	100,3 ±1,6	104,4 ±4,1	104,5 ±3,5	114,4
B3O7								
B3O8	53,6 ±1,1	70,8 ±1,3	83,2 ±6,4	96,6 ±1,4	103,1 ±2,8	111,0 ±1,6		
B3O9								
