# Supplementary material for: Inter-laboratory agreement on embryo classification and clinical decision: Conventional morphological assessment vs. time lapse
Source: PLoS One. 2017 Aug 25;12(8):e0183328. doi: 10.1371/journal.pone.0183328 (PMC5571938; doi:10.1371/journal.pone.0183328)
Supplement: S3 Table — Majority response (percentage of centres giving the majority response). PN:Pronuclei, NC:No consensus. (PDF) [file pone.0183328.s003.pdf]

S3 Table. Morphological characteristics and clinical decision of the embryos analysed with EmbryoScope.

Embryoscope	Number PN	Size PN	Opposition 2PN	Direct division 1 to 3 cells	Direct division 3 to 5 cells	False cleavage	Asymmetry
B1O1	2 (100%)	Symmetrical (88.9%)	Yes (66.7%)	No (77.8%)	No (66.7%)	No (55.6%)	Asymmetry (100%)
B1O2	2 (100%)	Symmetrical (100%)	Yes (77.8%)	No (100%)	No (100%)	No (100%)	Symmetry (77.8%)
B1O3	2 (100%)	Symmetrical (88.9%)	Yes (77.8%)	No (100%)	No (100%)	No (100%)	Symmetry (100%)
B1O4	2 (100%)	Symmetrical (100%)	Yes (77.8%)	No (77.8%)	No (66.7%)	Yes (55.6%)	Symmetry (88.9%)
B1O5	2 (66.6%)	Asymmetrycal (85.7%)	Yes (85.7%)	No (100%)	No (100%)	No (60%)	Symmetry (100%)
B1O6	2 (100%)	Symmetrical (100%)	Yes (88.9%)	No (100%)	No (100%)	No (88.9%)	Symmetry (88.9%)
B1O7	2 (100%)	Symmetrical (100%)	Yes (88.9%)	No (100%)	No (100%)	No (100%)	Symmetry (100%)
B1O8	2 (100%)	Symmetrical (100%)	Yes (88.9%)	Yes (77.8%)	No (100%)	Yes (88.9%)	Asymmetry (77.8%)
B1O9	2 (100%)	Symmetrical (88.9%)	Yes (77.8%)	No (100%)	No (100%)	No (100%)	Asymmetry (88.9%)
B1O10	2 (100%)	Symmetrical (88.9%)	Yes (88.9%)	No (100%)	Yes (88.9%)	No (100%)	Asymmetry (77.8%)
B1O11	2 (100%)	Symmetrical (100%)	Yes (88.9%)	No (55.6%)	Yes (100%)	Yes (88.9%)	Asymmetry (66.7%)
B1O12	2 (100%)	Symmetrical (100%)	Yes (87.5%)	Yes (62.5%)	No (100%)	No (83.3%)	Asymmetry (83.3%)
B2O1	0 (100%)						
B2O2	2 (100%)	Symmetrical (100%)	Yes (88.9%)	No (100%)	No (100%)	No (100%)	Asymmetry (88.9%)
B2O3	2 (100%)	Symmetrical (100%)	Yes (77.8%)	No (100%)	No (100%)	No (100%)	Symmetry (88.9%)
B2O4	2 (100%)	Symmetrical (88.9%)	Yes (88.9%)	No (100%)	No (100%)	No (100%)	Symmetry (77.8%)
B2O5	2 (100%)	Symmetrical (100%)	Yes (77.8%)	No (77.8%)	No (100%)	Yes (77.8%)	Asymmetry (75.0%)
B2O6	0 (100%)						
B2O7	2 (100%)	Symmetrical (100%)	Yes (88.9%)	Yes (77.8%)	Yes (57.1%)	No (100%)	Asymmetry (62.5%)
B2O8	2 (100%)	Symmetrical (100%)	Yes (88.9%)	No (100%)	No (55.6%)	No (77.8%)	Asymmetry (75.0%)
B2O9	2 (100%)	Symmetrical (100%)	Yes (88.9%)	No (100%)	No (100%)	No (100%)	Asymmetry (66.7%)
B2O10	2 (100%)	Symmetrical (88.9%)	Yes (88.9%)	No (100%)	No (100%)	No (100%)	Symmetry (88.9%)
B3O1	2 (100%)	Symmetrical (100%)	Yes (87.5%)	No (100%)	No (100%)	No (100%)	Asymmetry (75.0%)
B3O2	2 (100%)	Symmetrical (100%)	Yes (75.0%)	No (100%)	No (87.5%)	No (100%)	Asymmetry (62.5%)
B3O3	2 (100%)	Symmetrical (100%)	Yes (87.5%)	No (100%)	No (100%)	No (100%)	Symmetry (75.0%)
B3O4	0 (100%)						
B305	2 (100%)	Symmetrical (87.5%)	Yes (75.0%)	No (100%)	No (100%)	No (100%)	Symmetry (87.5%)
B3O6	2 (100%)	Symmetrical (100%)	Yes (75.0%)	No (100%)	No (100%)	No (62.5%)	Asymmetry (87.5%)
B3O7	2 (100%)	Symmetrical (100%)	Yes (87.5%)	No (100%)	No (100%)	No (75.0%)	Symmetry (85.7%)
B3O8	2 (100%)	Symmetrical (100%)	Yes (87.5%)	No (100%)	No (100%)	No (100%)	Symmetry (87.5%)
B3O9	2 (100%)	Symmetrical (100%)	Yes (87.5%)	No (100%)	No (100%)	No (100%)	Symmetry (62.5%)

S3 Table. Morphological characteristics and clinical decision of the embryos analysed with EmbryoScope (continuation).

Embryoscope	Fragmentation	Multinucleation	Vacuoles	Quality Day 2	Quality Day 3	Quality Day 5	Decision
B1O1	>10-25% (50%)	Absence (100%)	Presence (88.9%)	C (88.9%)	C (77.8%)	D (85.7%)	Discard (100%)
B1O2	>10-25% (55.6%)	Absence (100%)	Absence (100%)	B (66.7%)	B (55.6%)	C (55.6%)	Cryopreserve (88.9%)
B1O3	≤10% (100%)	Absence (100%)	Absence (100%)	A (55.7%)	A (55.7%)	C (57.1%)	Cryopreserve (55.6%)
B1O4	≤10% (100%)	Presence (61.1%)	Presence (55.6%)	D (77.8%)	D (88.9%)	D (100%)	Discard (100%)
B1O5	≤10% (100%)	Absence (100%)	Absence (100%)	D (100%)	D (100%)	D (100%)	Discard (100%)
B1O6	≤10% (100%)	Absence (100%)	Absence (100%)	A (100%)	D (44.4%)	D (100%)	Discard (100%)
B1O7	>10-25% (88.9%)	Absence (100%)	Absence (100%)	B (66.7%)	B (66.7%)	B (66.7%)	Transfer (55.6%)
B1O8	≤10% (100%)	Absence (100%)	Presence (77.7%)	NC (33.3%)	NC (44.4%)	C (55.6%)	Cryopreserve (55.6%)
B1O9	>10-25% (66.7%)	Absence (100%)	Absence (88.9%)	NC (33.3%)	C (55.6%)	B (44.4%)	Cryopreserve (55.6%)
B1O10	≤10% (100%)	Absence (100%)	Presence (66.7%)	C (55.6%)	B (55.6%)	D (100%)	Discard (100%)
B1O11	>10-25% (77.8%)	Absence (100%)	Absence (88.9%)	B (44.4%)	D (77.8%)	D (100%)	Discard (100%)
B1O12	26-35% (42.9%)	Absence (90.9%)	Presence (57.1%)	D (62.5%)	D (75.0%)	D (100%)	Discard (100%)
B2O1							
B2O2	≤10% (88.9%)	Absence (83.3%)	Absence (66.7%)	NC (33.3%)	B (44.4%)	C (44.4%)	Cryopreserve (77.8%)
B2O3	>10-25% (66.7%)	Absence (100%)	Presence (77.7%)	B (75.0%)	NC (50.0%)	C (50.0%)	Cryopreserve (100%)
B2O4	>10-25% (56.6%)	Absence (100%)	Presence (100%)	A (66.7%)	C (44.4%)	D (44.4%)	Cryopreserve (55.6%)
B2O5	≤10% (100%)	Absence (81.25%)	Absence (100%)	A (66.7%)	NC (44.4%)	C (44.4%)	Cryopreserve (66.7%)
B2O6							
B2O7	≤10% (100%)	Absence (93.3%)	Absence (100%)	C (37.5%)	C (62.5%)	D (100%)	Discard (100%)
B2O8	>10-25% (75.0%)	Absence (93.3%)	Absence (100%)	C (44.4%)	C (55.6%)	D (100%)	Discard (100%)
B2O9	≤10% (88.9%)	Absence (100%)	Absence (100%)	A (56.6%)	B (66.7%)	A (88.9%)	Transfer (100%)
B2O10	≤10% (88.9%)	Absence (100%)	Absence (100%)	A (66.7%)	A (56.6%)	C (55.6%)	Cryopreserve (88.9%)
B3O1	≤10% (100%)	Absence (93.8%)	Presence (87.5%)	NC (37.5%)	A (50.0%)	B (50.0%)	Cryopreserve (100%)
B3O2	≤10% (75.0%)	Absence (100%)	Presence (62.5%)	NC (37.5%)	C (50.0%)	C (50.0%)	Cryopreserve (75.0%)
B3O3	≤10% (87.5%)	Absence (93.8%)	Absence (87.5%)	A (62.5%)	NC (37.5%)	C (87.5%)	Cryopreserve (62.5%)
B3O4							
B305	≤10% (100%)	Absence (100%)	Presence (87.5%)	A (75%)	A (75%)	C (50.0%)	Cryopreserve (100%)
B3O6	≤10% (75.0%)	Absence (93.3%)	Presence (62.5%)	NC (50%)	C (87.5%)	D (83.3%)	Discard (100%)
B3O7	≤10% (100%)	Absence (100%)	Presence (62.5%)	A (87.5%)	NC (37.5%)	A (50.0%)	Transfer (75.0%)
B3O8	≤10% (100%)	Absence (100%)	NC (50%)	A (87.5%)	A (87.5%)	A (50.0%)	Cryopreserve (87.5%)
B3O9	≤10% (87.5%)	Absence (100%)	Absence (62.5%)	A (50.0%)	A (50.0%)	A (50.0%)	Cryopreserve (75.0%)
