# Supplementary material for: Inter-laboratory agreement on embryo classification and clinical decision: Conventional morphological assessment vs. time lapse
Source: PLoS One. 2017 Aug 25;12(8):e0183328. doi: 10.1371/journal.pone.0183328 (PMC5571938; doi:10.1371/journal.pone.0183328)
Supplement: S4 Table — Majority response (percentage of centres giving the majority response). PN:Pronuclei, NC:No consensus. (PDF) [file pone.0183328.s004.pdf]

S4 Table. Morphological characteristics and clinical decision of the embryos analysed with Primo Vision.

Primo Vision	Number PN	Size PN	Opposition 2PN	Direct division 1 to 3 cells	Direct division 3 to 5 cells	False cleavage	Asymmetry	Fragmentation
B1O1	2 (100%)	Yes (93,75%)	Symmetrical (100%)	No (100%)	No (100%)	No (100%)	Symmetry (76.9%)	≤10% (100%)
B1O2	0 (100%)							
B1O3	2 (100%)	Yes (87.5%)	Symmetrical (87.5%)	No (100%)	No (100%)	No (100%)	Symmetry (78.6%)	≤10% (100%)
B1O4	2 (100%)	Yes (87.5%)	Symmetrical (100%)	No (57.1%)	No (57.1%)	No (64.3%)	Asymmetry (61.5%)	≤10% (71.4%)
B1O5	0 (100%)							
B1O6	2 (100%)	Yes (87.5%)	Symmetrical (93.8%)	No (100%)	No (92.9%)	No (100%)	Symmetry (92.9%)	≤10% (92.9%)
B1O7	2 (100%)	Yes (81.3%)	Symmetrical (75%)	No (93.3%)	No (64.3%)	No (100%)	Symmetry (57.4%)	≤10% (92.8%)
B1O8	2 (100%)	Yes (75%9	Symmetrical (100%)	No (71.4%)	No (85.7%)	No (57.1%)	Asymmetry (85.7%)	NC (46.7%)
B1O9	2 (100%)	Yes (81.3%)	Symmetrical (100%)	No (71.4%)	No (64.3%)	No (92.9%)	Asymmetry (71.4%)	≤10% (66.6%)
B1O10	2 (100%)	Yes (87.5%)	Symmetrical (100%)	No (57.1%)	No (71.4%)	No (60.0%)	Asymmetry (53.9%)	≤10% (71.4%)
B1O11	2 (100%)	Yes (87.5%)	Symmetrical (100%)	No (57.1%)	No (71.4%)	No (60.0%)	NC (50%)	≤10% (71.4%)
B1O12	2 (100%)	Yes (93.8%)	Symmetrical (87.5%)	No (100%)	No (100%)	No (85.7%)	Symmetry (100%)	≤10% (100%)
B1O13	2 (100%)	Yes (93.8%)	Symmetrical (93.8%)	No (100%)	No (85.7%)	No (100%)	Asymmetry (85.7%)	≤10% (73.3%)
B1O14	2 (100%)	Yes (87.5%)	Symmetrical (100%)	No (100%)	No (92.9%)	No (64.3%)	Asymmetry (85.7%)	≤10% (85.7%)
B2O1	2 (100%)	Yes (86.7%)	Symmetrical (86.7%)	No (100%)	No (100%)	No (100%)	Symmetry (100%)	≤10% (100%)
B2O2	2 (100%)	Yes (93.3%)	Symmetrical (100%)	No (78.6%)	No (100%)	No (85.7%)	Asymmetry (61.5%)	>35% (93.3%)
B2O3	0 (90.9%)							
B2O4	2 (87.5%)	Yes (92.9%)	Symmetrical (100%)	No (100%)	No (92.3%)	No (53.9%)	Asymmetry (92.3%)	>35% (57.1%)
B2O5	2 (100%)	Yes (93.3%)	Symmetrical (100%)	No (100%)	No (100%)	No (71.4%)	Symmetry (71.43%)	26-35% (46.7%)
B2O6	2 (100%)	Yes (86.7%)	Symmetrical (86.7%)	No (76.9%)	No (100%)	Yes (100%)	Asymmetry (92.9%)	NC (33.3%)
B2O7	2 (100%)	Yes (93.3%)	Symmetrical (87.5%)	No (100%)	No (100%)	No (78.6%)	Symmetry (100%)	≤10% (92.9%)
B2O8	2 (87.5%)	Yes (69.2%)	Symmetrical (78.6%)	No (100%)	No (100%)	No (100%)	Asymmetry (78.6%)	≤10% (53.3%)
B2O9	2 (100%)	Yes (93.3%)	Symmetrical (100%)	No (100%)	No (100%)	No (78.6%)	Symmetry (78.6%)	≤10% (64.3%)
B2O10	2 (100%)	Yes (93.3%)	Symmetrical (100%)	No (100%)	No (85.7%)	No (76.9%)	Asymmetry (57.1%)	≤10% (57.2%)
B2O11	2 (100%)	Yes (86.7%)	Symmetrical (81.3%)	Yes (71.4%)	No (71.4%)	No (76.9%)	Asymmetry (86.7%)	26-35% (60.0%)
B2O12	2 (100%)	Yes (86.7%)	Symmetrical (87.5%)	No (92.9%)	No (85.7%)	No (57.1%)	NC (50%)	>10-25% (60%)
B3O1	2 (100%)	Yes (93.3%)	Symmetrical (100%)	No (100%)	No (100%)	No (100%)	Symmetry (85.7%)	≤10% (100%)
B3O2	2 (100%)	Yes (93.3%)	Symmetrical (100%)	No (100%)	No (100%)	No (71.4%)	Symmetry (57.1%)	NC (50%)
B3O3	2 (100%)	Yes (93.3%)	Symmetrical (87.5%)	No (100%)	No (100%)	No (100%)	Symmetry (100%)	≤10% (92.9%)
B3O4	2 (100%)	Yes (86.7%)	Symmetrical (100%)	No (100%)	No (100%)	No (100%)	Symmetry (100%)	≤10% (92.9%)
B305	2 (100%)	Yes (93.3%)	Symmetrical (100%)	No (92.9%)	No (100%)	No (100%)	Symmetry (85.7%)	≤10% (100%)
B3O6	2 (100%)	Yes (80.0%)	Symmetrical (100%)	No (100%)	No (100%)	No (100%)	Symmetry (78.6%)	≤10% (95.7%)
B3O7	0 (100%)							
B3O8	2 (100%)	Yes (93.3%)	Symmetrical (100%)	No (100%)	No (100%)	No (100%)	Symmetry (85.7%)	≤10% (92.9%)
B3O9	2 (93.3%)	Yes (60.0%)	Symmetrical (85.7%)	No (100%)	No (100%)	Yes (75.0%)	Symmetry (100%)	NC (50%)

S4 Table. Morphological characteristics and clinical decision of the embryos analysed with Primo Vision (continuation).

Primo Vision	Multinucleation	Vacuoles	Quality Day 2	Quality Day 3	Quality Day 5	Decision
B1O1	Absence (74.1%)	Absence (100%)	A (53.3%)	NC (33.3%)	C (66.7%)	Cryopreserve (60%)
B1O2						
B1O3	Absence (88.9%)	Absence (100%)	A (73.3%)	A (40.0%)	D (66.7%)	Discard (92.9%)
B1O4	Absence (74.1%)	Absence (92.9%)	B (40.0%)	B (40.0%)	D (92.9%)	Discard (100%)
B1O5						
B1O6	Absence (92.9%)	Absence (100%)	A (92.9%)	A (85.7.9%)	D (78.6%)	Discard (92.3%)
B1O7	NC (50.0%)	Absence (71.4%)	B (46.7%)	B (40.0%)	A (53.3%)	Cryopreserve (66.7%)
B1O8	Absence (60.7%)	Presence (93.3%)	D (73.3%)	D (73.3%)	D (100%)	Discard (100%)
B1O9	Absence (92.9%)	Absence (100%)	B (73.3%)	B (66.7%)	C (60.0%)	Cryopreserve (93.3%)
B1O10	Absence (88.9%)	Presence (78.5%)	C (60%)	C (60%)	D (86.7%)	Discard (100%)
B1O11	Absence (92.9%)	Absence (100%)	B (66.7%)	D (64.3%)	D (66.7%)	Discard (71.4%)
B1O12	Absence (72.4%)	Absence (93.3%)	A (64.3%)	A (64.3%)	A (35.7%)	Transfer (50.0%)
B1O13	Absence (69.0%)	Absence (53.3%)	B (46.7%)	B (46.7%)	C (40.0%)	Cryopreserve (80.0%)
B1O14	Absence (62.1%)	Absence (93.3%)	NC (28.7%)	NC (28.7%)	D (57.1%)	Discard (61.5%)
B2O1	Absence (96.6%)	Presence (78.6%)	A (80.0%)	A (73.3%)	A (40.0%)	Transfer (53.3%)
B2O2	Absence (96.2%)	Absence (92.3%)	D (66.7%)	D (80.0%)	D (100%)	Discard (92.3%)
B2O3						
B2O4	Absence (92.3%)	Absence (61.5%)	D (50.0%)	D (71.4%)	D (100%)	Discard (100%)
B2O5	Absence (89.3%)	Presence (93.4%)	C (46.7%)	C (60.0%)	C (53.3%)	Discard (53.9%)
B2O6	NC (50.0%)	Presence (64,3%)	D (93.3%)	D (100%)	D (100%)	Discard (100%)
B2O7	Absence (75.9%)	Absence (53.3%)	A (66.7%)	A (60%)	C (46.7%)	Cryopreserve (71.4%)
B2O8	Absence (92.9%)	Presence (86,7%)	A (53.3%)	C (53.3%)	D (100%)	Discard (100%)
B2O9	Absence (89.3%)	Absence (86.7%)	B (33.3%)	B (53.3%)	NC (33.3%)	Cryopreserve (53.3%)
B2O10	Absence (82.1%)	Absence (78.6%)	C (53.3%)	C (53.3%)	D (66.7%)	Discard (78.6%)
B2O11	Absence (100%)	Absence (64.3%)	D (60.0%)	D (53.3%)	D (53.3%)	Discard (85.7%)
B2O12	Absence (92.3%)	Presence (73.3%)	B (50.0%)	B (50.0%)	C (64.3%)	Cryopreserve (84.6%)
B3O1	Absence (62.1%)	Absence (85.7%)	A (40.0%)	A (53.3%)	C (40.0%)	Cryopreserve (64.3%)
B3O2	Presence (53.6%)	Absence (64.3%%)	NC (26.7%)	NC (33.3%)	D (80%)	Discard (92.9%)
B3O3	Absence (82.1%)	Absence (78.6%)	A (86.7%)	A (46.7%)	A (46.7%)	Cryopreserve (73.3%)
B3O4	Absence (100%)	Absence (100%)	A (80.0%)	A (46.7%)	A (60.0%)	Transfer (60.0%)
B305	Absence (96.4%)	Absence (100%)	A (66.7%)	A (60.0%)	B (40.0%)	Cryopreserve (80.0%)
B3O6	Absence (55.2%)	Absence (92.9%)	A (40.0%)	NC (33.3%)	A (40.0%)	Cryopreserve (73.3%)
B3O7						
B3O8	Presence (62.1%)	Absence (100%)	A (40.0%)	NC (33.3%)	A (53.3%)	Cryopreserve (85.7%)
B3O9	Absence (100%)	Absence (100%)	D (100%)	D (100%)	D (100%)	Discard (100%)
