# Supplementary material for: Inter-laboratory agreement on embryo classification and clinical decision: Conventional morphological assessment vs. time lapse
Source: PLoS One. 2017 Aug 25;12(8):e0183328. doi: 10.1371/journal.pone.0183328 (PMC5571938; doi:10.1371/journal.pone.0183328)
Supplement: S5 Table — Majority response (percentage of centres giving the majority response).NC: No consensus. (PDF) [file pone.0183328.s005.pdf]

S5 Table. Morphological characteristics of the Day 2 and Day 3 embryos analysed with CMA.

			Day 2		
CMA	Asymmetry	Fragmentation	Multinucleation	Vacuoles	Classification
B1O1/13	Symmetry (100%)	≤10% (92.3%)	Abscense (98,9%)	Abscense (100%)	A (87.2%)
B1O2/13	Symmetry (89,7%)	≤10% (100%)	Abscense (100%)	Abscense (100%)	A (82.1%)
B1O3/13	Symmetry (100%)	≤10% (100%)	Abscense (100%)	Abscense (100%)	A (89.7%)
B1O4/13	Symmetry (89,7%)	≤10% (92.3%)	Abscense (87.2%)	Abscense (100%)	A (51.3%)
B1O5/13	Symmetry (92,1%)	≤10% (94.7%)	Abscense (100%)	Abscense (100%)	A (71.5%)
B2O1/13	Symmetry (87,2%)	≤10% (56,4%)	Abscense (97.4%)	Abscense (100%)	B (43.6%)
B2O2/13	Asymmetry (97,3%)	>10-25% (76.9%)	Abscense (100%)	Abscense (97.4%)	C (61.5%)
B2O3/13	Symmetry (86,8%)	≤10% (100%)	Abscense (100%)	Abscense (94.9%)	B (71.8%)
B2O4/13	Symmetry (97,4%)	≤10% (59,0%)	Abscense (100%)	Abscense (100%)	B (48.7%)
B2O5/13	Symmetry (61,5%)	>25-36% (51.3%)	Abscense (100%)	Abscense (100%)	C (57.9%)
B3O1/13	Asymmetry (91,9%)	>10-25% (47.4%)	Abscense (100%)	Abscense (100%)	C (92.1%)
B3O2/13	Symeetry (56,4%)	≤10% (97.4%)	Abscense (64.1%)	Abscense (100%)	B (38.5%)
B3O3/13	Symmetry (97,4%)	>10-25% (71.1%)	Abscense (100%)	Abscense (100%)	B (79.5%)
B3O4/13	Asymmetry (66,7%)	>36% (89.2%)	Abscense (97.1%)	Abscense (100%)	D (78.4%)
B3O5/13	Symmetry (78,9%)	≤10% (100%)	Abscense (100%)	Abscense (100%)	D (56.4%)
B1O1/14	Symmetry (51,4%)	≤10% (100%)	Abscense (89.2%)	Abscense (100%)	NC (33.3%)
B1O2/14	Symmetry (82,1%)	≤10% (97.4%)	Abscense (94.9%)	Abscense (100%)	A (71.8%)
B1O3/14	Symmetry (84,6%)	≤10% (76.9%)	Abscense (100%)	Abscense (100%)	A (66.7%)
B1O4/14	Symmetry (97,4%)	≤10% (100%)	Abscense (66.7%)	Abscense (92.3%)	A (56.4%)
B1O5/14	Symmetry (94,9%)	≤10% (100%)	Abscense (100%)	Abscense (100%)	A (92.3%)
B2O1/14	Asymmetry (89,5%)	≤10% (100%)	Abscense (100%)	Abscense (100%)	C (97.4%)
B2O2/14	Asymmetry (97,4%)	≤10% (81.6%)	Abscense (81.6%)	Abscense (92.1%)	C (71.1%)
B2O3/14	Symmetry (68,4%)	≤10% (100%)	Abscense (94.7%)	Abscense (94.7%)	B (62.6%)
B2O4/14	Symmetry (100%)	≤10% (100%)	Abscense (100%)	Abscense (100%)	B (65.8%)
B2O5/14	Asymmetry (97,4%)	>10-25% (73.7%)	Abscense (97.4%)	Abscense (86.8%)	C (52.6%)
B3O1/14	Asymmetry (81,6%)	≤10% (100%)	Abscense (100%)	Abscense (100%)	C (86.8%)
B3O2/14	Asymmetry (100%)	>10-25% (71.1%)	Abscense (100%)	Abscense (97.4%)	C (97.4%)
B3O3/14	Symmetry (100%)	≤10% (100%)	Abscense (97.4%)	Abscense (97.4%)	B (94.8%)
B3O4/14	Symmetry (81,6%)	≤10% (71.1%)	Abscense (100%)	Abscense (97.3%)	A (39.5%)
B3O5/14	Symmetry (73,7%)	≤10% (100%)	Abscense (100%)	Abscense (97.4%)	A (65.8%)

S5 Table. Morphological characteristics of the Day 2 and Day 3 embryos analysed with CMA (continuation).

	Day 3				
CMA	Asymmetry	Fragmentation	Multinucleation	Vacuoles	Classification
B1O1/13	Symmetry (89,7%)	≤10% (97.4%)	Abscense (97.4%)	Abscense (97.4%)	B (60.5%)
B1O2/13	Symmetry (94,9%)	≤10% (100%)	Abscense (100%)	Abscense (100%)	A (89.7%)
B1O3/13	Symmetry (94,9%)	≤10% (100%)	Abscense (100%)	Abscense (100%)	A (84.6%)
B1O4/13	Asymmetry (59,0%)	≤10% (92.3%)	Abscense (97.4%)	Abscense (100%)	C (59.0%)
B1O5/13	Symmetry (81,6%)	≤10% (92.1%)	Abscense (100%)	Abscense (100%)	A (52.6%)
B2O1/13	Asymmetry (97,3%)	>10-25% (71.1%)	Abscense (86.8%)	Abscense (100%)	C (44.7%)
B2O2/13	Asymmetry (63,9%)	>10-25% (68.4%)	Abscense (100%)	Abscense (55.3%)	C (60.5%)
B2O3/13	Symmetry (81,6%)	≤10% (100%)	Abscense (100%)	Abscense (100%)	B (73.7%)
B2O4/13	Asymmetry (88,9%)	≤10% (66,7%)	Abscense (97.2%)	Abscense (100%)	C (91.7%)
B2O5/13	Symmetry (82,9%)	>25-36% (42,9%)	Abscense (100%)	Abscense (100%)	C (51.4%)
B3O1/13	Asymmetry (71,4%)	>36% (37,9%)	Abscense (96.4%)	Abscense (100%)	D (85.7%)
B3O2/13	Symmetry (86,1%)	≤10% (97.3%)	Abscense (100%)	Abscense (100%)	D (94.4%)
B3O3/13	Symmetry (70,3%)	>25-36% (71.8%)	Abscense (100%)	Abscense (97.4%)	D (89.5%)
B3O4/13	Symmetry (71,9%)	>36% (93.8%)	Abscense (100%)	Abscense (100%)	D (83.3%)
B3O5/13	Symmetry (94,6%)	≤10% (100%)	Abscense (100%)	Abscense (100%)	C (54.1%)
B1O1/14	Symmetry (97,4%)	≤10% (100%)	Abscense (100%)	Abscense (100%)	A (59.0%)
B1O2/14	Symmetry (89,7%)	≤10% (97,4%)	Abscense (97.4%)	Abscense (100%)	A (71.8%)
B1O3/14	Symmetry (53,8%)	≤10% 66,7%)	Abscense (81.6%)	Abscense (97.4%)	B (38.5%)
B1O4/14	Symmetry (69,2%)	≤10% (100%)	Abscense (94.9%)	Abscense (100%)	A (50.0%)
B1O5/14	Symmetry (100%)	≤10% (100%)	Abscense (97.4%)	Abscense (100%)	A (94.7%)
B2O1/14	Symmetry (55,3%)	≤10% (100%)	Abscense (100%)	Abscense (100%)	C (73.7%)
B2O2/14	Asymmetry (81,1%)	≤10% (78,9%)	Abscense (100%)	Abscense (97.4%)	C (67.6%)
B2O3/14	Asymmetry (55,3%)	≤10% (100%)	Abscense (100%)	Abscense (100%)	B (54.1%)
B2O4/14	Asymmetry (81,6%)	≤10% (94,7%)	Abscense (94.7%)	Abscense (94.4%)	C (92.1%)
B2O5/14	Asymmetry (73,7%)	>25-36% (71.1%)	Abscense (97.4%)	Abscense (92.1%)	C (43.2%)
B3O1/14	Asymmetry (89,5%)	≤10% (100%)	Abscense (97.4%)	Abscense (100%)	D (92.1%)
B3O2/14	Asymmetry (84,2%)	>25-36% (84.2%)	Abscense (100%)	Abscense (97.4%)	D (70.3%)
B3O3/14	Symmetry (100%)	≤10% (97,3%)	Abscense (100%)	Abscense (100%)	D (97.3%)
B3O4/14	Symmetry (60,5%)	≤10% (71,1%)	Abscense (100%)	Abscense (100%)	C (27.0%)
B3O5/14	Symmetry (89,5%)	≤10% (100%)	Abscense (100%)	Abscense (100%)	D (97.4%)
