# Supplementary material for: Inter-laboratory agreement on embryo classification and clinical decision: Conventional morphological assessment vs. time lapse
Source: PLoS One. 2017 Aug 25;12(8):e0183328. doi: 10.1371/journal.pone.0183328 (PMC5571938; doi:10.1371/journal.pone.0183328)
Supplement: S6 Table — Majority response (percentage of centres giving the majority response). (PDF) [file pone.0183328.s006.pdf]

S6 Table. Morphological classification and clinical decision of the Day 5 embryos analysed with CMA.

CMA	Classification	Decision
B4O1/13	B (45.5%)	Cryopreserve (69.2%)
B4O2/13	B (51.5%)	Cryopreserve (66.7%)
B4O3/13	B (63.6%)	Cryopreserve (84.6%)
B4O4/13	B (43.8%)	Cryopreserve (74.4%)
B4O5/13	B (35.3%)	Cryopreserve (56.4%)
B5O1/13	B (57.1%)	Transfer (71.8%)
B5O2/13	B (61.1%)	Cryopreserve (74.4%)
B5O3/13	C (54.3%)	Cryopreserve (53.9%)
B5O4/13	C (42.9%)	Cryopreserve (55.3%)
B5O5/13	C (75.0%)	Cryopreserve (63.16%)
B4O1/14	B (66.7%)	Cryopreserve (68.4%)
B4O2/14	B (48.4%)	Cryopreserve (60.0%)
B4O3/14	A (41.4%)	Transfer (62.2%)
B4O4/14	B (60.7%)	Cryopreserve (77.8%)
B4O5/14	C (55.6%)	Cryopreserve (75.7%)
B5O1/14	C (71.9%)	Cryopreserve (48.7%)
B5O2/14	C (73.5%)	Cryopreserve (50.0%)
B5O3/14	C (54.6%)	Discard (67.6%)
B5O4/14	C (44.4%)	Transfer (83.8%)
B5O5/14	D (56.3%)	Discard (84.2%)
